# Simvastatin and atorvastatin reduce the mechanical properties of tendon constructs in vitro and introduce catabolic changes in the gene expression pattern

**DOI:** 10.1371/journal.pone.0172797

**Published:** 2017-03-06

**Authors:** Pernilla Eliasson, Rene B. Svensson, Antonis Giannopoulos, Christian Eismark, Michael Kjær, Peter Schjerling, Katja M. Heinemeier

**Affiliations:** 1 Institute of Sports Medicine Copenhagen, Dept of Orthopedic Surgery M, Bispebjerg Hospital and Center for Healthy Aging, Faculty of Health and Medical Sciences, University of Copenhagen, Copenhagen, Denmark; 2 Department of Clinical and Experimental Medicine, Linköping University, Linköping, Sweden; University of Rochester, UNITED STATES

## Abstract

Treatment with lipid-lowering drugs, statins, is common all over the world. Lately, the occurrence of spontaneous tendon ruptures or tendinosis have suggested a negative influence of statins upon tendon tissue. But how statins might influence tendons is not clear. In the present study, we investigated the effect of statin treatment on mechanical strength, cell proliferation, collagen content and gene expression pattern in a tendon-like tissue made from human tenocytes in vitro. Human tendon fibroblasts were grown in a 3D tissue culture model (tendon constructs), and treated with either simvastatin or atorvastatin, low or high dose, respectively, for up to seven days. After seven days of treatment, mechanical testing of the constructs was performed. Collagen content and cell proliferation were also determined. mRNA levels of several target genes were measured after one or seven days. The maximum force and stiffness were reduced by both statins after 7 days (p<0.05), while the cross sectional area was unaffected. Further, the collagen content was reduced by atorvastatin (p = 0.01) and the cell proliferation rate was decreased by both types of statins (p<0.05). Statin treatment also introduced increased mRNA levels of MMP-1, MMP-3, MMP-13, TIMP-1 and decreased levels of collagen type 1 and 3. In conclusion, statin treatment appears to have a negative effect on tendon matrix quality as seen by a reduced strength of the tendon constructs. Further, activated catabolic changes in the gene expression pattern and a reduced collagen content indicated a disturbed balance in matrix production of tendon due to statin administration.

## Introduction

Statin medication is commonly used all over the world [1]. Statins are lipid-lowering drugs that inhibit 3-hydroxy-3-methylglutaryl coenzyme a (HMG-CoA) reductase, which normally turns HMG-CoA into mevalonic acid. Statin treatment is known to have relatively few side-effects, but skeletal muscle weakness and muscle pain are among the fairly common side-effects reported [2]. It is not completely known why these side-effects appear, but it is likely a cholesterol independent effect.

During the last 15 years, an increasing attention has also been raised to potential side effects by statin treatment on tendons, with tendinopathy or ruptures as the consequence. There are case-reports that describe spontaneous tendon ruptures during statin use [3–5]. A few retrospective studies and one case-control study [2, 5–8] also indicate that there might be a connection between statin treatment and tendon complications. However the connection is far from established, nor is there any clear mechanistic explanation for this coupling. Tendon pain or ruptures have been shown to appear relatively soon after statin medication has been introduced, and the pain appears to disappear again after the treatment has been stopped [8]. It has been reported that 50% of all the cases occur during the first year of treatment.

The retrospective studies have suggested that different statins could be more or less prone to have adverse effects in relation to tendons. Rosuvastatin and atorvastatin have been more frequently linked to side effects than other statins [2, 7, 8], while simvastatin gives rise to mixed outcomes [6, 7, 9]. Two retrospective studies indicate that simvastatin might have a protective role against tendinopathy, especially in patients suffering from severe hyperlipidemia [6, 7, 9] while another study indicates that simvastatin treatment is linked to tendon complications [8].

Rat studies have shown that statin treatment appears to have a detrimental effect on the mechanical properties of intact Achilles tendons and it is inducing biochemical changes in the tendon [10–12]. However, statin treatment during tendon or ligament healing appears to have a positive effect [13–15], especially on tendon to bone healing [13, 15]. Statins have been suggested to destabilize cell membranes, lead to tenocyte apoptosis or disturb matrix remodeling through an altered expression and activity of MMPs and collagens [8, 16]. Statins can also influence cell migration [13, 17, 18] and cell proliferation by arresting the cells in the G1-phase [18]. However the results on cell proliferation are again contradictive. They are also suggested to have an anti-fibrotic effect and this might explain a positive effect on muscle tissue fibrosis in shoulders with rotator-cuff tear [19].

The results from the retrospective studies, animal studies, and cell culture studies are not conclusive on whether statin treatment in fact is detrimental for tendon tissue or not. Also there are suggestions for the mechanism of action but this is still not clear. Since hypercholesterolemia is a potential risk factor for tendon adversities it is preferable to study the effect of statins on human tendon cells without the influence of this, in order to study the pure mechanism of action of statins on human tendon cells. Tendon cells within a tendon exist in a three-dimensional (3D) extracellular matrix (ECM); however, the in vitro studies on statins so far have used cells in 2D monolayers. We have recently introduced a fibrin based 3D culture system for primary adult tendon fibroblasts, referred to as tendon constructs [20–23]. This system is ideal to investigate collagen formation in vitro because all the collagen has been produced by the fibroblasts in contrast to collagen based 3D models. The constructs will also increase in strength with time in culture [21]. The effect of statin treatment in general, and of simvastatin in particular, on tendons and tendon fibroblasts is somewhat contradictive.

The aim of this study was therefore to investigate if two different statins, simvastatin and atorvastatin, influenced human tendon cells and extracellular matrix, in a 3D culture model, similar to a real tissue. The aim was also to study if statin treatment induced any alterations in the gene expression pattern in these cells.

## Materials and methods

### Cell culture

Tendon fibroblast were isolated from human semitendinosus and gracilis tendons, from patients undergoing reconstructive surgery after anterior cruciate ligament (ACL) rupture. Briefly, six patients (18–32 years old) gave written informed consent and the experiments with human tissue were approved by the local ethical committee (den nationale videnskabsetiske komité, ref. H-3-2010-070). The investigation has been conducted according to the Declaration of Helsinki. The tissue was collected during surgery and immediately transported to a cell culture laboratory. Muscle tissue remnants were carefully scraped off and the tendon tissue was cut into small pieces ~2 mm^2^. The tissue was digested overnight in DMEM/F12 (Gibco, Invitrogen) supplemented with 0.1% collagenase type II (Worthington) and 20% fetal bovine serum (FBS, Gibco, Invitrogen). Cells were seeded out in flasks the following day and cultured to confluence in DMEM/F12 supplemented with 10% FBS. The method has previously been described in detail [23]. The cells used for these experiments were either in their 3rd or 4th passage.

### Tendon construct formation

Tendon constructs from human tenocytes were assembled as previously described with small modifications [23]. Briefly, six-well plates were coated with SYLGARD (Dow-Chemicals) at the bottom. Two anchors, short silk sutures (0.5 cm, Ethicon) were pinned onto the SYLGARD with minutien insect pins (0.1 mm diameter, Fine Science Tools GmbH) at a distance of 10 mm apart. After sterilization of the plates (by ethanol immersion), 250 000 cells were plated within a fibrin gel (800 μl) and quickly spread on top of the SYLGARD. The fibrin gel consisted of human tendon fibroblasts in DMEM/F12 containing 10% FBS, 4 mg/ml human fibrinogen, 10 μg/ml aprotenin and 1 unit of human thrombin (all Sigma Aldrich). The fibrin gel was allowed to set for 60 minutes at 37°C before it was covered with DMEM/F12 (supplemented with 10% FBS, 0.2 mM L-ascorbic acid 2-phosphate, 0.05 mM L-Proline and 1% Penicillin-Streptomycin). The medium was replaced every second to third day and adhesions to the side of the well were detached using a fine pipette tip to allow gel contraction. After 8–14 days the cells contracted the structure to a rod-like structure in between the anchor points.

### Statin treatment of the constructs

After 14 days, when all constructs were contracted, statin treatment was introduced. The constructs were treated with two different types of statins, simvastatin (Sigma Aldrich, S6196) and atorvastatin (Sigma Aldrich, P20001). Both statins were dissolved in DMSO and sterile filtered before use. The stock solution was thereafter diluted in cell culture medium DMEM/F12 supplemented with 5% FBS, 0.2 mM L-ascorbic acid 2-phosphate and 0.05 mM L-Proline. The statins were used in two different doses 0.05μM and 0.5μM. As controls, since the statins were dissolved in DMSO, constructs were also treated with DMSO (0.02%, which correspond to the dose DMSO seen in 0.5μM treatments) or cell culture medium alone. The constructs were treated with statins for 1 or 7 days and the effect of statin treatment was evaluated by mechanical testing, hydroxyproline assay for collagen content, MTT assay for cell proliferation and gene expression analysis by qPCR.

### Mechanical testing of the constructs

**Fig 1:**
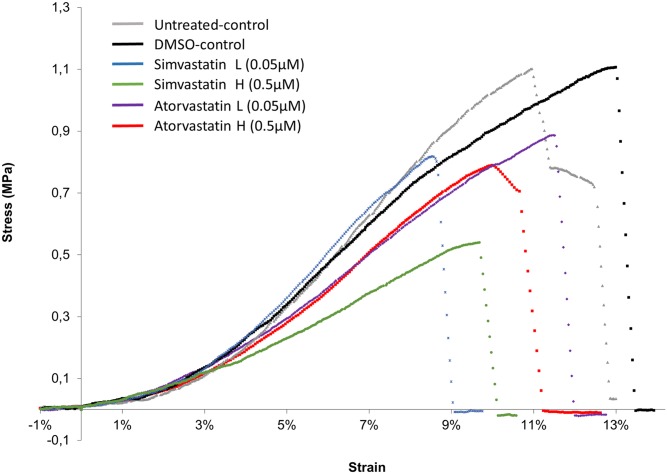
Stress-strain curves during mechanical testing. Representative curves from each treatment group (all from the same cell line). Grey is untreated-controls, black is DMSO-controls, blue is low dose simvastatin treatment (0.05μM), green is high dose simvastatin treatment (0.5μM), purple is low dose atorvastatin treatment (0.05μM) and red is high dose atorvastatin treatment (0.5μM). The samples treated with high dose simvastatin had a significantly lower maximum stress compared to the DMSO-control.

Tensile testing of the constructs was performed after 7 days of statin treatment (21 days after cell seeding). The testing was performed in a PC-driven micromechanical rig with a liquid chamber (20 N load-cell, sampling rate 10 Hz; Deben, Suffolk, UK). A stereoscopic microscope (SMZ1000, Nikon, Tokyo, Japan) with C-mount lens (8x), equipped with a 15 Hz digital camera (DFWX700, Sony, Tokyo, Japan; 640x480 Pixel) was used for imaging during the test to verify clamping length and monitor the rupture site of the construct. The tendon constructs were glued on specimen plates with a mounting distance of 12.5 mm. The specimen was thereafter transferred to a cell culture medium-bath and after a short adaptation period, the test was started. The samples were stretched at 4 mm/min until failure (Fig 1). Construct diameter and mounting length were measured before the stretching started by capturing images though the microscope and calculating the distances using ImageJ (NIH, USA). The diameter was measured four different places (the thickest and thinnest places on the right and left side of the construct using 4x objective magnification) and an average cross sectional area was calculated assuming a circular cross-section. The length of the samples was also measured at 0.8x objective magnification. Force was filtered by a running average over 10 data points (equal to 1 s or ~0.5% strain) before calculating stress based on the cross sectional area. Strain was determined from the length at the onset of force (point where stress first exceeds 10 kPa). Tensile modulus was calculated as the peak slope determined by linear regression over a 1% strain range. The mechanical testing was performed in triplicates for each cell line and a mean value for each parameter across these triplicates was used for further analysis. For technical reasons, three of the samples only had duplicate mechanical measurements. The mean for each cell line treated with statins was thereafter used for statistical analysis.

### Hydroxyproline assay

Following mechanical testing, tendon constructs were snap frozen in liquid nitrogen and stored at -80°C until further analysis. One sample from each cell line and treatment was used for collagen content assay. The samples were freeze-dried (48 hours) and weighed using an ultra-microbalance scale (Mettler-Toledo GmBH, Gießen, Germany) in a humidity and temperature controlled room. The samples were hydrolysed in 6 M HCl at 110°C for 18 h, thereafter dried at 95°C, and washed by adding water and drying 3 times. The samples were dissolved again in 600 μl acetate-citrate buffer and 75 μl chloramine-T solution was added to 150 μl of the samples followed by incubation for 20 min at room temperature. 75 μl aldehyde-perchloric acid solution was added and further incubated at 60°C for 25 min. This procedure has been described more in detail elsewhere [22]. The reaction was stopped by placing the samples on ice and the absorbance was measured at a wavelength of 570 nm. The samples were correlated with a standard curve from pure hydroxyproline (Sigma, H1637) and collagen content values were calculated using the sample dry weight assuming a hydroxyproline content of 13.3% by mass [24].

### MTT-assay

An MTT assay was used for quantification of cell viability/cell proliferation in the constructs, after 7 days of statin treatment. Cell culture medium with 10% of MTT (Sigma Aldrich) was added in each well with constructs. The constructs were incubated for 2 hours in the incubator before each construct was transferred to 1 ml of DMSO. The samples were then incubated for 15 minutes at room temperature on an orbital shaker (protected from light). After 15 minutes, all crystals were dissolved and 100 μl of the DMSO solution was used for absorbance reading.

### Quantitative real-time PCR

The mRNA expression of different gene targets was measured after 1 or 7 days of statin treatment using quantitative real-time reverse transcriptase (RT) PCR. Targets and primer sequences are provided in Table 1. Tendon constructs were quickly rinsed in PBS before harvesting and thereafter transferred to RNAse free tubes containing 1 ml TriReagent (Molecular Research Centre, Cincinnati, OH, USA), 5 stainless steel beads (2.3 mm in diameter) and 5 silicon-carbide sharp particles (1 mm in diameter) for mechanical disruption (BioSpec Products, Inc., Bartlesville, Oklahoma, USA). Samples were mechanically disrupted using a FastPrep^®^-24 instrument (MP Biomedicals, Inc., Illkirch, France), and subsequently 100 μl bromo-chloropropane (Molecular Research Centre) was added in order to separate the samples into an aqueous and an organic phase. 120 μg/ml of glycogen was added to each sample to improve RNA yield. Following isolation of the aqueous phase, RNA was precipitated using isopropanol, washed in ethanol and dissolved in RNAse-free water. RNA concentrations were determined by spectroscopy at 260 nm and RNA quality was confirmed by gel electrophoresis.

**Table 1:** PCR primers.

Target	Sense	Antisense
RPLP0	GGAAACTCTGCATTCTCGCTTCCT	GCTCCTTGCCGAGAAGCAGAAC
GAPDH	CCTCCTGCACCACCAACTGCTT	GAGGGGCCATCCACAGTCTTCT
COL1A1	GGCAACAGCCGCTTCACCTAC	GCGGGAGGACTTGGTGGTTTT
COL3A1	CACGGAAACACTGGTGGACAGATT	ATGCCAGCTGCACATCAAGGAC
SCX	CAGCCCAAACAGATCTGCACCTT	CTGTCTTTCTGTCGCGGTCCTT
TNMD	GAAGCGGAAATGGCACTGATGA	TGAAGACCCACGAAGTAGATGCCA
EGR-1	GCAGCCCTACGAGCACCTGACC	AACTGGTCTCCACCAGCACCTTC
EGR-2	CCTTTGACCAGATGAACGGAGTG	TAGGTGCAGAGACGGGAGCAAA
MMP-1	CGAATTTGCCGACAGAGATGAAG	GGGAAGCCAAAGGAGCTGTAGATG
MMP-2	CCGCCTTTAACTGGAGCAAAAACA	TTGGGGAAGCCAGGATCCATTT
MMP-3	GATCCTGCTTTGTCCTTTGATGCTGT	CTGAGGGATTTGCGCCAAAAGTG
MMP-9	AGCGAGGTGGACCGGATGTT	AGAAGCGGTCCTGGCAGAAATAG
MMP-13	CCTGATGACGATGTACAAGGGA	TGGCATCAAGGGATAAGGAAGGG
TIMP-1	CGGGGCTTCACCAAGACCTACA	TGGTCCGTCCACAAGCAATGA
HSP-27	GCTGACGGTCAAGACCAAGGATG	TGAAGCACCGGGAGATGTAGCC
HSP-70	GTGGCTGGACGCCAACACCTT	TTACACACCTGCTCCAGCTCCTTC

500 ng of RNA was transcribed to complementary DNA (cDNA) by using Omniscript reverse transcriptase (Qiagen, Hilden, Germany). For each target gene, 5 μl of 20x diluted cDNA (in 10mM Tris, 1 mM EDTA buffer, pH 8 with 1 ng/μl salmon DNA) was amplified in 25 μl Quantitect SYBR Green Master Mix (Qiagen) with specific primers (100 nM each) on a real-time PCR machine (MX3000P, Stratagene, La Jolla, CA, USA). The thermal profile was 95°C, 10 min → (95°C, 15 s → 58°C, 30 s → 63°C, 90 s) × 50 → 95°C, 60 s → 55°C, 30 s → 95°C, 60 s. Signal intensity was acquired at the 63°C step, and the threshold cycle (Ct) values were related to a standard curve made with the cloned PCR product. Specificity was confirmed by melting curve analysis after amplification (the 55°C to 95°C step). The large ribosomal protein P0 (RPLP0) mRNA was chosen as internal control for normalization. To test RPLP0 mRNA stability, another common mRNA control was also measured, Glyceraldehyde 3-phosphate dehydrogenase (GAPDH). The GAPDH/RPLP0 ratio appeared to be stable (no significant difference between the groups and RPLP0 was chosen for normalization.

### Statistical analysis

The mechanical data, the hydroxyproline assay and the MTT assay were first tested with a Wilcoxon matched-pairs signed rank test to test if there were any statistical difference between DMSO-controls and untreated-controls. The treatment effect of statins was thereafter analyzed with a repeated measures Friedman test followed by Dunn´s test for multiple testing against the DMSO-controls. These statistical analyses were done in GraphPad Prism.

Log transformed gene expression data was first analyzed by comparing if there was any difference between DMSO-controls or untreated-controls with a repeated measures 2-way ANOVA with the day 1 and 7 data from DMSO-controls and untreated-controls. When no difference was found we continued to normalize all the statin treated samples to the same day DMSO-control from each cell line and thereafter analyzed the data again with a repeated measures 2-way ANOVA with time and treatment as independent variables. This was followed by Dunnett´s method for multiple comparisons against the DMSO-controls. One sample in the untreated-control group (day 1) was excluded from analyses on all targets due to an extreme deviating result. The MMP-1 expression was more than 400 standard deviations from the mean after normalization within the cell line. Also the expression of TIMP-1, MMP-9 and scleraxis had all greater values than 2 standard deviations from the mean and there was no detectable expression of tenomodulin. This exclusion was done before the statistical analyses were performed. p<0.05 was considered significant. These statistical analyses were done in SigmaPlot.

## Results

There was no difference between untreated-controls and DMSO-controls in the mechanical data, the hydroxyproline assay or the MTT assay. The statistical analyses have therefore been done comparing DMSO-controls with the different statin treatments.

### Simvastatin and atorvastatin decreased the mechanical strength of the constructs

**Fig 2:**
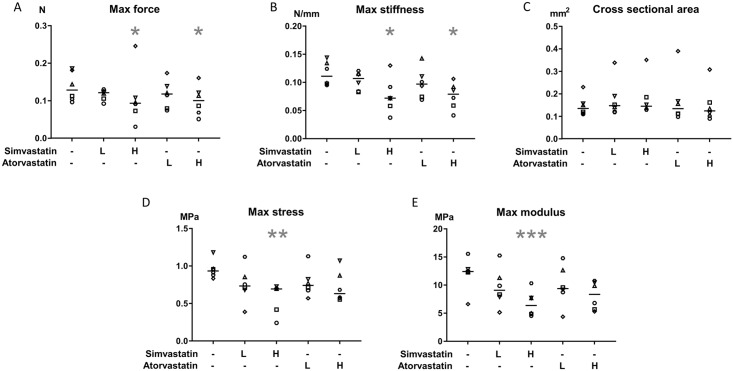
Construct mechanical data. Maximum force, maximum stiffness, cross sectional area, maximum stress and maximum modulus of tendon constructs with or without statin treatment for 7 days. Low dose (L) statin treatment corresponds to 0.05μM (simvastatin or atorvastatin) and high dose (H) corresponds to 0.5μM. Significant changes from DMSO-control are indicated by * (p<0.05), ** (p<0.01) or *** (p<0.001). The line represents median value. n = 6 different donor-specific cell lines, where each symbol represent the mean value from three replicates from one cell line. The force, stiffness, stress and modulus were all reduced after 7 days of high dose simvastatin treatment. High dose treatment with atorvastatin also reduced the force and stiffness and tended to reduce the maximum modulus (p = 0.07).

The maximum force was reduced by approximately 30% by both high dose simvastatin (0.5 μM) and high dose atorvastatin compared to DMSO-controls (p<0.05 for both, Fig 2, Table 2). The maximum stiffness was also reduced by both high dose treatments by approximately 40% (p<0.05 for both). The cross sectional area was unaffected by treatment, and thereby the material properties were altered with a reduced maximum stress by 33% in the high dose simvastatin group and the modulus was reduced by 38% (p<0.01 and p<0.001 respectively). High dose treatment with atorvastatin tended to reduce the maximum modulus by 25% (p = 0.07) but the maximum stress was not significantly different from DMSO-controls (28% lower in atorvastatin treated samples compared to DMSO-controls, p = 0.11). Energy to failure, deformation at max force and strain at max stress were unaffected by treatment.

**Table 2:** Mechanical data.

	Untreated-control	DMSO-control	Simvastatin low (0.05 μM)	Simvastatin high (0.5μM)	Atorvastatin low (0.05 μM)	Atorvastatin high (0.5μM)
Cross-sectional area (mm^2^)	0.14 (0.11–0.27)	0.14 (0.11–0.23)	0.15 (0.12–0.34)	0.15 (0.13–0.35)	0.13 (0.10–0.39)	0.12 (0.09–0.31)
Max force (N)	0.14 (0.10–0.23)	0.13 (0.10–0.19)	0.12 (0.09–0.13)	0.09 (0.03–0.25)*	0.12 (0.07–0.17)	0.10 (0.05–0.16)*
Max stiffness (N/mm)	0.11 (0.08–0.15)	0.11 (0.10–0.14)	0.11 (0.08–0.12)	0.07 (0.04–0.13) *	0.10 (0.07–0.14)	0.08 (0.04–0.11) *
Max stress (MPa)	0.87 (0.73–1.45)	0.93 (0.83–1.18)	0.73 (0.39–1.12)	0.69 (0.24–0.73) **	0.74 (0.57–1.31)	0.63 (0.55–1.07)
Max modulus (MPa)	9.78 (7.15–17.5)	12.4 (6.62–15.6)	9.09 (5.16–15.3)	6.35 (4.53–10.3) ***	9.38 (4.37–14.8)	8.34 (5.30–10.8)
Deformation at max force (mm)	2.03 (1.87–2.45)	1.89 (1.40–2.73)	1.86 (1.74–2.14)	1.83 (1.16–2.75)	1.87 (1.42–2.74)	2.00 (1.38–2.39)
Strain at max stress (%)	14.2 (13.0–18.7)	13.1 (9.76–18.4)	12.9 (12.3–14.9)	12.2 (7.58–20.3)	13.4 (10.3–18.9)	13.6 (9.16–16.7)
Energy to failure (MJ/m^3^)	0.12 (0.08–0.24)	0.10 (0.06–0.23)	0.10 (0.07–0.12)	0.08 (0.02–0.32)	0.08 (0.05–0.23)	0.09 (0.04–0.21)

Mechanical properties on test to failure in tendon constructs with or without statin treatment for 7 days, median (range), n = 6 different donor-specific cell lines.

Significant changes from DMSO-control are indicated by:

* p<0.05,

** p<0.01 or

*** p<0.001.

### The collagen content was reduced by high dose atorvastatin treatment

**Fig 3:**
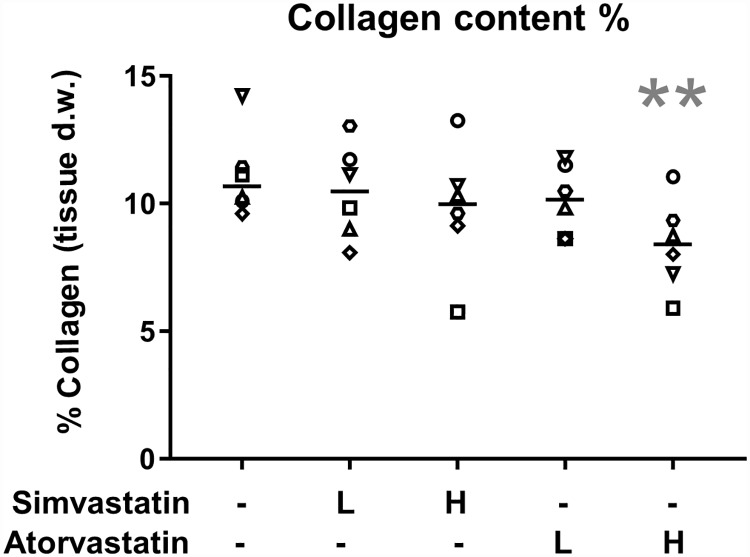
Collagen quantification. Total collagen content in tendon constructs with or without statin treatment for 7 days. Low dose (L) statin treatment corresponds to 0.05μM (simvastatin or atorvastatin) and high dose (H) corresponds to 0.5μM. The line represents median value. Individual donor-specific cell lines are represented by the six different symbols. The collagen content was approximately 10% of the construct dry weight. High dose treatment with atorvastatin reduced the collagen content compared to DMSO-controls, this was not seen after simvastatin treatment. Significant changes from DMSO-control are indicated by ** (p = 0.01).

The collagen content was approximately 10% of the tendon construct dry weight (ranging from 5.8–14.2%, Fig 3). The collagen content was reduced by 22% after 7 days of high dose atorvastatin treatment (p = 0.01). Neither high dose simvastatin nor low dose atorvastatin treatment influenced the collagen content.

### Cell proliferation was decreased with high dose statin treatment

**Fig 4:**
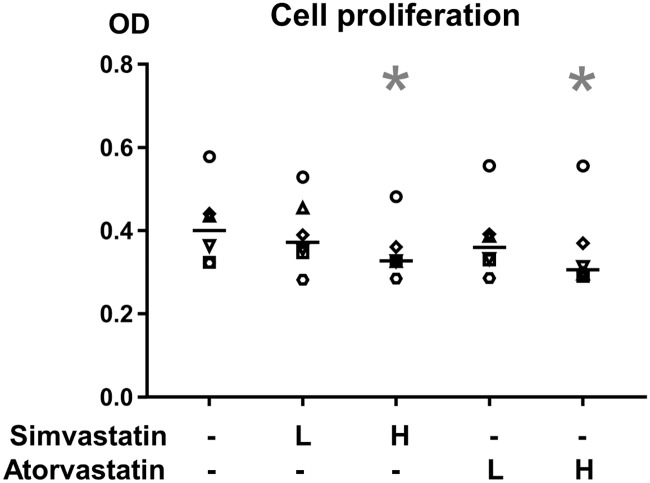
Cell proliferation. Cell proliferation in tendon constructs, with or without statin treatment for 7 days, measured by a MTT assay. The data is presented as optical density (OD) value. Low dose (L) statin treatment corresponds to 0.05μM (simvastatin or atorvastatin) and high dose (H) corresponds to 0.5μM. The line represents median value. Individual donor-specific cell lines are represented by the six different symbols. The cell proliferation was reduced in constructs treated with high dose simvastatin or high dose atorvastatin. Significant changes from DMSO-control are indicated by * (p<0.05).

Seven days of high dose statin treatment (both simvastatin and atorvastatin) reduced the relative cell number by 14% compared to DMSO-controls (p<0.05 for both, Fig 4). Low dose treatment did not affect the proliferation during this treatment time.

### There was an increased gene expression of MMPs and a decreased expression of collagens

The immediate gene expression response (after 1 day of treatment) and the delayed gene expression response (after 7 days of treatment) were analyzed on several gene targets (Table 1).

We first tested if there was any effect of 0.02% DMSO or time by using a repeated measures two-way ANOVA (DMSO-control vs untreated-control at day 1 and 7). There was no significant effect of DMSO treatment except for a slightly decreased expression of collagen type 3 (p<0.05). In the DMSO-controls there was an effect of time on two genes, tenomodulin and TIMP-1. Tenomodulin was 8-fold increased between day 1 and day 7 and (p<0.001) and TIMP-1 was at the same time decreased by 12% (p<0.001) (S1 Fig).

**Fig 5:**
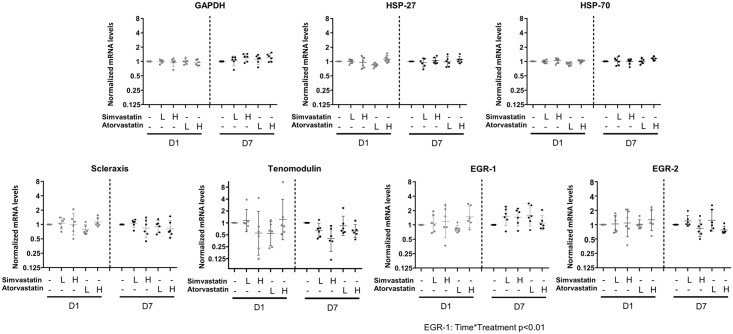
Gene expression analysis: Effect of treatment on GAPDH, heat shock proteins and tendon related genes. Gene expression after 1 or 7 days of statin treatment. The samples were analyzed for GAPDH, heat shock proteins and tendon related genes. Low dose (L) statin treatment corresponds to 0.05μM of either simvastatin or atorvastatin and high dose (H) corresponds to 0.5μM. The DMSO-control is set as baseline, and mRNA expression in constructs treated with statins are shown relative to DMSO-control for each cell line and day. Data is presented on a logarithmic y scale as geometric means ± 95% confidence interval (CI). Individual donor-specific cell lines are represented by dots. Significant interaction in the repeated measures 2-way ANOVA (time*treatment) are below each graph.

**Fig 6:**
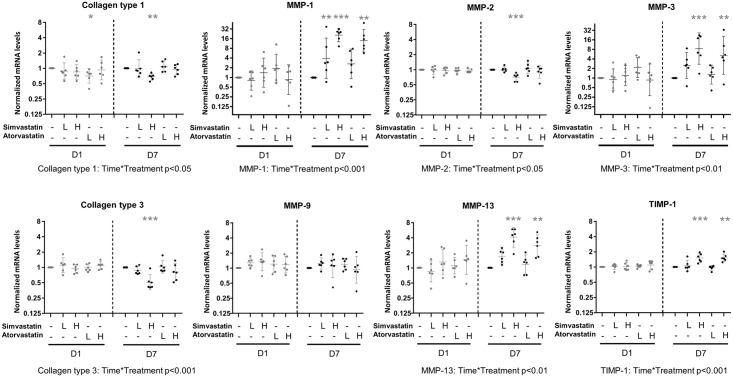
Gene expression analysis: Effect of treatment on collagen, MMPs and TIMP-1. Gene expression after 1 or 7 days of statin treatment. The samples were analyzed for collagens, MMPs and TIMP-1. Low dose (L) statin treatment corresponds to 0.05μM of either simvastatin or atorvastatin and high dose (H) corresponds to 0.5μM. The DMSO-control is set as baseline, and mRNA expression in constructs treated with statins are shown relative to DMSO-control for each cell line and day. Significant changes from DMSO-control are indicated by * (p<0.05), ** (p<0.01), and *** (p<0.001). Data is presented on a logarithmic y scale as geometric means ± 95% confidence interval (CI). Individual donor-specific cell lines are represented by dots. Significant interaction in the repeated measures 2-way ANOVA (time*treatment) are indicated below each graph.

All the data was thereafter normalized to the DMSO-control for each cell line and treatment day and analyzed with a repeated measures 2-way ANOVA with time and treatment as independent variables. There was a significant interaction (treatment x day) for collagen type 3, MMP-1 and TIMP-1 (p<0.001 for all) MMP-3, MMP-13, and EGR-1 (p<0.01 for all) and collagen type 1 and MMP-2 (p<0.05 for both) (Figs 5 and 6 and S2 and S3 Figs). There was no interaction but a general treatment effect on HSP-27 and HSP-70 and a general time effect on tenomodulin. The expression of GAPDH, EGR-2 and scleraxis were all unaffected by both time and treatment.

One day of statin treatment did not appear to really influence the gene expression of the selected genes (only a small decrease in the expression of collagen type 1 by low dose atorvastatin treatment).

Seven days of high dose treatment (0.5μM) with simvastatin reduced the expression of collagen type 1 with 32% (p<0.01) and the expression of collagen type 3 was reduced by 49% (p<0.001, Fig 6). Also the MMP-2 expression was reduced by 30% (p<0.001). The other MMPs had an increased expression after treatment (MMP-1, MMP-3, MMP-13 and TIMP-1, p<0.001 for all). The expression of MMP-1 was 20 times higher in the simvastatin treated group compared to the DMSO-controls and MMP-3 was 8-times higher. High dose atorvastatin for 7 days also induced changes in the gene expression pattern with a higher expression of MMP-1 (13 times higher compared to DMSO-controls), MMP-3, MMP-13 and TIMP-1 (p<0.01 for all). Seven days of low dose treatment with simvastatin (0.05μM) induced a higher expression of MMP-1 (p<0.01). There was also a tendency for an increased expression of MMP-1 after low dose treatment with atorvastatin for seven days (p = 0.06). All other targets remained at similar levels as DMSO-controls after treatment with low doses of statins.

## Discussion

High cholesterol, and treatment thereof with statins, is becoming more and more common all over the world. In the present study we demonstrated that statin treatment had a negative effect on tendon matrix quality in a tendon construct model as seen by a reduced strength and stiffness of the tendon constructs after 7 days of treatment. In association with this, statins resulted in activation of catabolic changes in the gene expression pattern of the tendon, and altered collagen content which indicated a disturbed balance in matrix production of the tendon due to statin administration.

Simvastatin and atorvastatin may have different potential for negative effects on tendons. Atorvastatin has been pointed out as one of the most harmful statins for tendon tissue [7] while simvastatin has appeared with mixed findings. Some studies indicate that simvastatin has a negative impact on tendon tissue, while other studies suggest that simvastatin might even have a positive effect. Both atorvastatin and simvastatin had a negative effect on the tendon constructs in our study, however, the effect of simvastatin was in fact stronger and more pronounced especially on the material properties of the tendon constructs and also with more and larger effects on the gene expression pattern. This indicates that simvastatin indeed is harmful for tendon tissue and its extracellular matrix. However, high dose atorvastatin treatment was the only treatment which had an effect on collagen content, and this was reduced by 22% compared to DMSO-controls, despite lack of change in cross sectional area, effect on peak stress and only a trend to an effect on modulus. Simvastatin on the other hand had no effect on the collagen content to our surprise. This indicates that there might be some differences in the mechanism of action on human tendon cells and extracellular matrix between these two statins. The range of the dosage of the two statins in clinical settings is similar, though the average dose for atorvastatin is slightly lower than the average dose for simvastatin. We used the same dose for both statins in our experiments. However, a previous study used somewhat different doses between these two statins, where atorvastatin was administered in a higher dose [17]. In spite of this difference in dosage, also this study showed that simvastatin had a more pronounced effect on cell migration compared to the higher dose atorvastatin [17]. Higher doses (10μM) of either of these two statins are toxic for tendon fibroblasts in vitro (previous unpublished data). The doses used in this study did not appear to be toxic even if the proliferation was slightly decreased with the high dose. A previous in vitro study has shown that the cells appear to arrest in the G1-phase of the cell cycle when treated with simvastatin, an effect that could be reversed by mevalonate or two down-stream intermediates [18]. There are however also studies which show no effect on proliferation by treatment with lovastatin [17] or even a higher proliferation with atorvastatin treatment [13]. The discrepancy between these results could perhaps be a question of timing or dosage. It was mainly the high dose, 0.5μM of either statin, that had an effect in these experiments. However, we treated the constructs for a maximum of seven days and a normal statin treatment, in patients, continues for years. The fact that just a short period of treatment had such a detrimental effect upon tendon tissue indicates that also long term adaptations may occur. We found that there is a need for a few days of treatment, in vitro, before we could see an effect. Thus, there was no effect on the immediate gene response, after one day of treatment.

Previous in vitro studies have only studied cells in a 2D culturing system and this has its limitations. We use a 3D culturing system with human tendon fibroblasts, which resembles more the normal environment for the tendon cells. Our model allows us to measure the amount of collagen that has been produced by the cells and we can also measure the mechanical properties of this tendon-like tissue as in animal experiments, but without the species differences. One additional advantage with this model is that we can also study the direct effect of statin treatment on human tendon cells without the influence of hypercholesterolemia, which in its turn can be harmful for tendon tissue [25, 26]. Statin treatment in our model appeared to decrease the mechanical properties of the tendon construct without influencing the cross-sectional area. Oral simvastatin or atorvastatin treatment in rats have also been shown to reduce the mechanical properties of the Achilles tendon without influencing the cross-sectional area, similar to our study [11, 12]. The lack of change in cross-sectional area and the lack of significantly altered total collagen levels after the treatment with simvastatin suggest that simvastatin could perhaps influence collagen cross-linking or collagen fibril size distribution giving a larger amount of smaller fibrils with weaker material properties. This would be in line with a recent study using the same tendon construct model, treating cells with a lysyl oxidase blocker to inhibit cross link formation over the exact same period as the present study. It was shown that mechanical properties were markedly changed in a similar way as in the present study, without observing any change in collagen content or tendon diameter [22]. It could however also be an effect of multiple different factors, each of which is not statistically significant, affecting the mechanics. The weaker matrix could also be an effect of increased MMP levels, which degrades the collagen and parts of this degraded collagen could still be measured by the hydroxyproline assay.

The mechanism for an adverse effect by statin treatment on tendons is not clear. MMPs have been suggested to have a potential role in this [8, 16]. So far, there is only one study which has investigated the effects of statins on two MMPs and showed that two months of statin treatment resulted in increased levels of MMP-2 and MMP-9 in the rat Achilles tendon [10]. This is in contrast to what we found with a decreased gene expression of MMP-2 and unchanged levels of MMP-9. Other MMPs have not been studied in tendon fibroblasts after statin treatment. We found that both simvastatin and atorvastatin increased the expression of MMP-1, MMP-3 and MMP-13. MMP-1 and MMP-13 are collagenases and are the main enzymes degrading collagen type I in tendons [27]. The increased mRNA levels could potentially lead to a weaker tendon matrix. The increased expression of MMP-1 is in contrast to studies on other cell types, e.g. simvastatin treatment have been shown to reduce the expression of MMP-1 in smooth muscle cells [28]. However, the expression of different MMPs and the MMP response can even differ a lot between different types of fibroblasts, depending on the origin [29]. Besides the increased MMP expression in this study, we also found a decreased expression of both collagen type 1 and 3. This has previously been shown after lovastatin treatment on human tendon fibroblasts in 2D culture, an effect that was assumed to be mediated via prenylation of GTPases [17]. Atorvastatin has also been shown to have a negative effect on collagen type 1 gene expression in rat Achilles tendons [10]. The general increase in MMPs and the decrease in collagen indicates that statin treatment leads to a catabolic gene expression pattern. We did not see any differences in the expression of tendon related genes, scleraxis, tenomodulin and EGR-1 and EGR-2, so statins do not appear to influence the cells in a different direction of differentiation. But we did see that tenomodulin was increased with time and development of the constructs. Also the expression of heat shock proteins remained the same after treatment.

There are some limitations with this study, we only measured the mRNA levels and not protein levels of most targets (with the exception of collagen). Also, when studying MMPs it is desirable to also measure the activity. This study is done in vitro and cells are altered as soon as you take them out from their normal environment. However, this is a 3D model that promotes the expression of tendon related genes, also seen in this study with an increased expression of tenomodulin with time. There are many different types of statins and we only tested two types in this study and the high levels of statins are perhaps not physiological. However this model enables direct assessment of how statins affect tendon cell behavior and tissue extracellular matrix without interference from the surroundings. Tendon constructs represents a “developing” tissue with an increasing amount of collagen formation in contrast to mature tendons in humans that have a relative static tissue after 18 years of age [30]. There were some discrepancies between treatment with simvastatin and atorvastatin, simvastatin had a more pronounced effect on the majority of the studied factors, besides the collagen content where only atorvastatin had a pronounced effect. The reason for this discrepancy is not know but it could indicate that the different drugs have different effects on the matrix.

## Conclusions

In conclusion, this study demonstrates that treatment with statins appear to negatively affect tendon cells and extracellular matrix. The biomechanical properties were decreased and the gene expression patterns were switched to a catabolic profile. This could be one reason why tendon injuries appear to be linked to statin treatment.

## Supporting information

S1 Fig Gene expression analysis: Effect of time. Normalized gene expression after 7 days of culture. The DMSO-controls from day 7 were all normalized to the corresponding DMSO-control at day 1 to illustrate the time effect. There was a significantly increased expression of tenomodulin from day 1 to day 7 and a reduced expression of TIMP-1 during this same time and this is indicated by *** (p<0.001). Data is presented on a logarithmic y scale as geometric means ± 95% confidence interval (CI). Individual donor-specific cell lines are represented by dots.
(TIF)

S2 Fig Gene expression analysis: Effect of treatment on GAPDH, heat shock proteins and tendon related genes. The gene expression after 1 or 7 days of statin treatment presented as the absolute values in arbitrary units (A.U.), which is a ratio between the mRNA and the housekeeping mRNA (RPLP0). Data is presented on a logarithmic y scale and the line represents the mean value. Individual donor-specific cell lines are represented by different symbols. Low dose (L) statin treatment corresponds to 0.05μM of either simvastatin or atorvastatin and high dose (H) corresponds to 0.5μM.
(TIF)

S3 Fig Gene expression analysis: Effect of treatment on collagen, MMPs and TIMP-1. The gene expression after 1 or 7 days of statin treatment presented as the absolute values in arbitrary units (A.U.), which is a ratio between the mRNA and the housekeeping mRNA (RPLP0). Data is presented on a logarithmic y scale and the line represents the mean value. Individual donor-specific cell lines are represented by different symbols. Low dose (L) statin treatment corresponds to 0.05μM of either simvastatin or atorvastatin and high dose (H) corresponds to 0.5μM. Significant changes from DMSO-control are indicated by * (p<0.05), ** (p<0.01), and *** (p<0.001).
(TIF)
