# Supplementary figures and images for: Simvastatin and atorvastatin reduce the mechanical properties of tendon constructs in vitro and introduce catabolic changes in the gene expression pattern
Source: PLoS One. 2017 Mar 6;12(3):e0172797. doi: 10.1371/journal.pone.0172797 (PMC5339395; doi:10.1371/journal.pone.0172797)

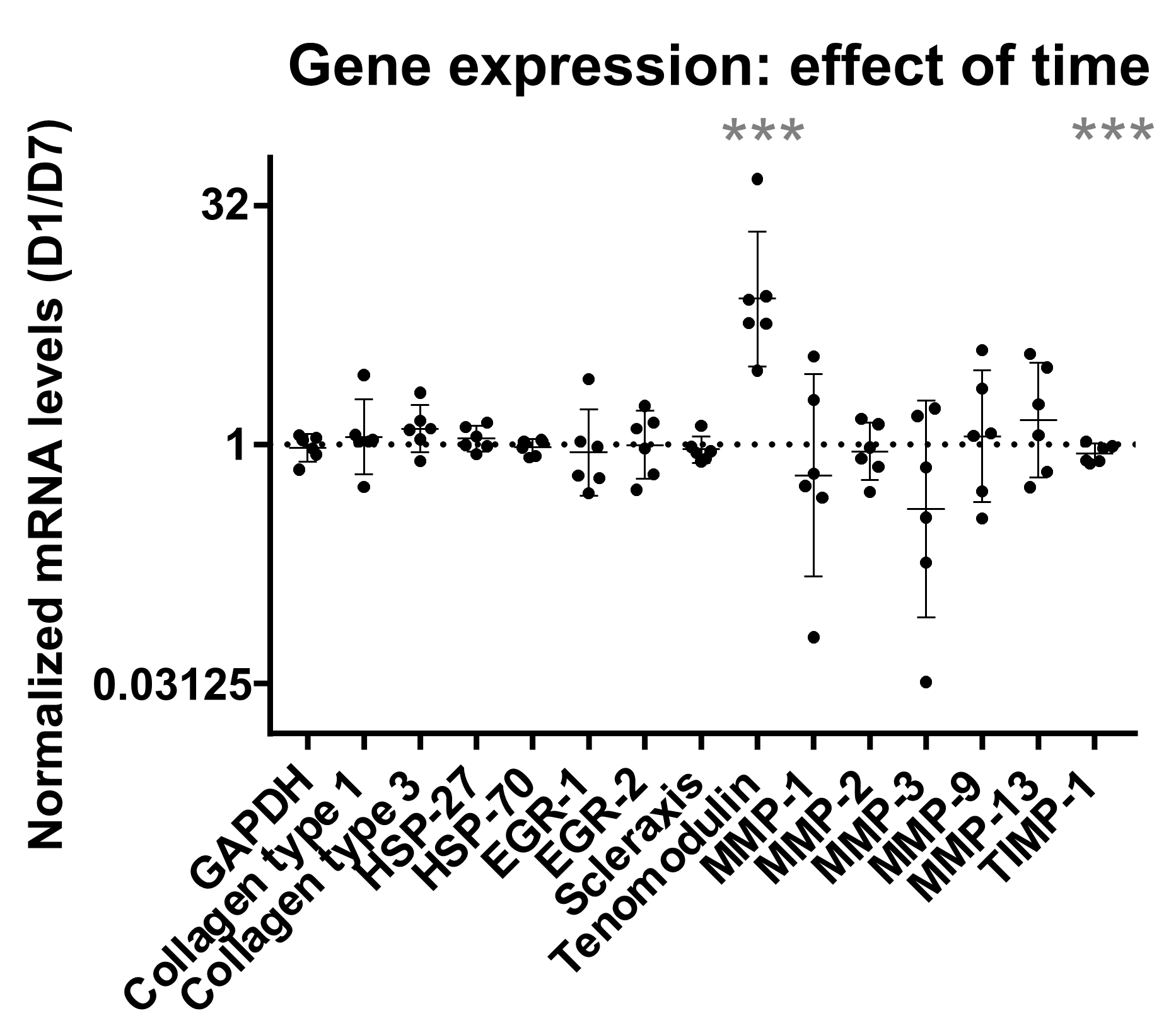

Supplement: S1 Fig — Normalized gene expression after 7 days of culture. The DMSO-controls from day 7 were all normalized to the corresponding DMSO-control at day 1 to illustrate the time effect. There was a significantly increased expression of tenomodulin from day 1 to day 7 and a reduced expression of TIMP-1 during this same time and this is indicated by *** (p<0.001). Data is presented on a logarithmic y scale as geometric means ± 95% confidence interval (CI). Individual donor-specific cell lines are represented by dots. (TIF) [file pone.0172797.s001.tif]

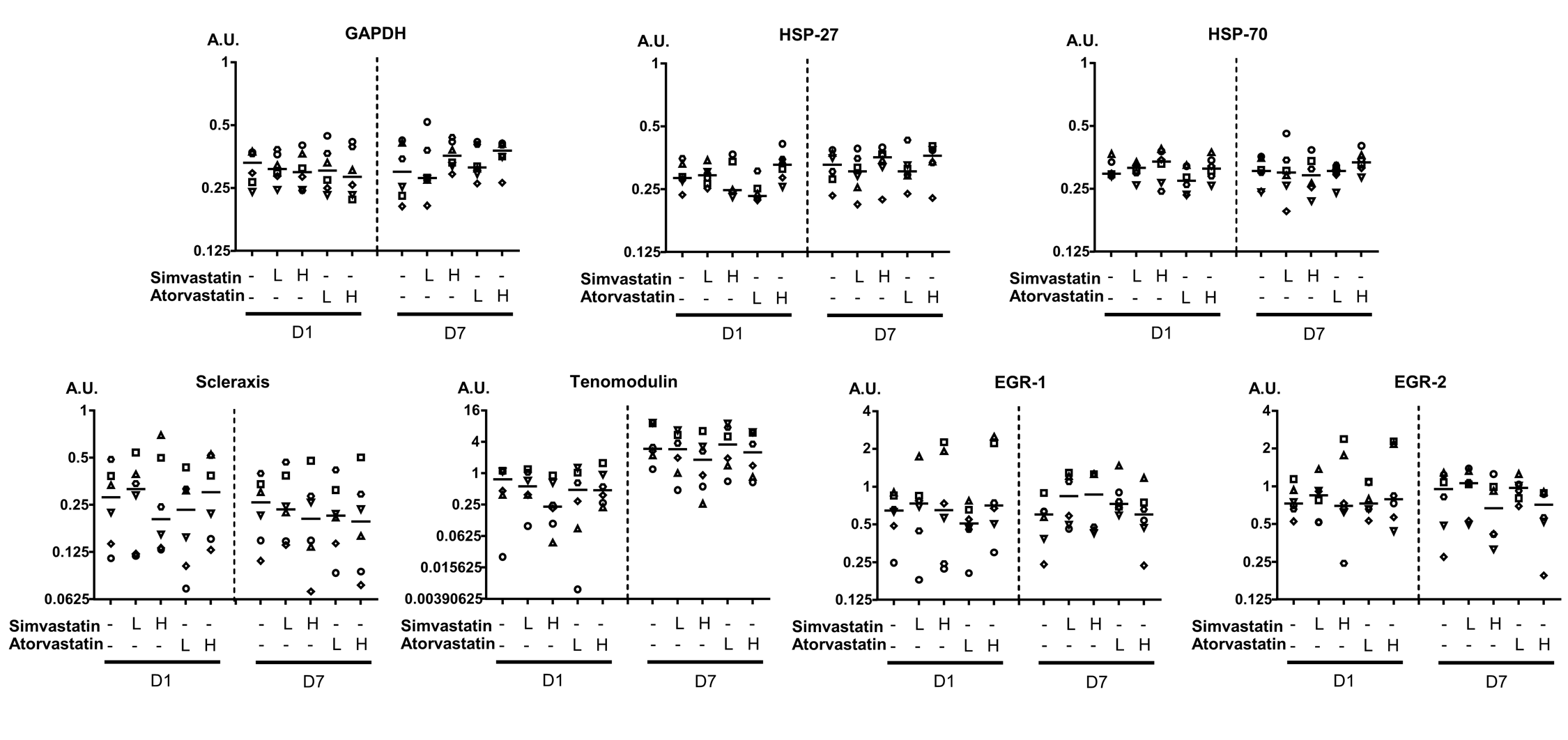

Supplement: S2 Fig — The gene expression after 1 or 7 days of statin treatment presented as the absolute values in arbitrary units (A.U.), which is a ratio between the mRNA and the housekeeping mRNA (RPLP0). Data is presented on a logarithmic y scale and the line represents the mean value. Individual donor-specific cell lines are represented by different symbols. Low dose (L) statin treatment corresponds to 0.05μM of either simvastatin or atorvastatin and high dose (H) corresponds to 0.5μM. (TIF) [file pone.0172797.s002.tif]

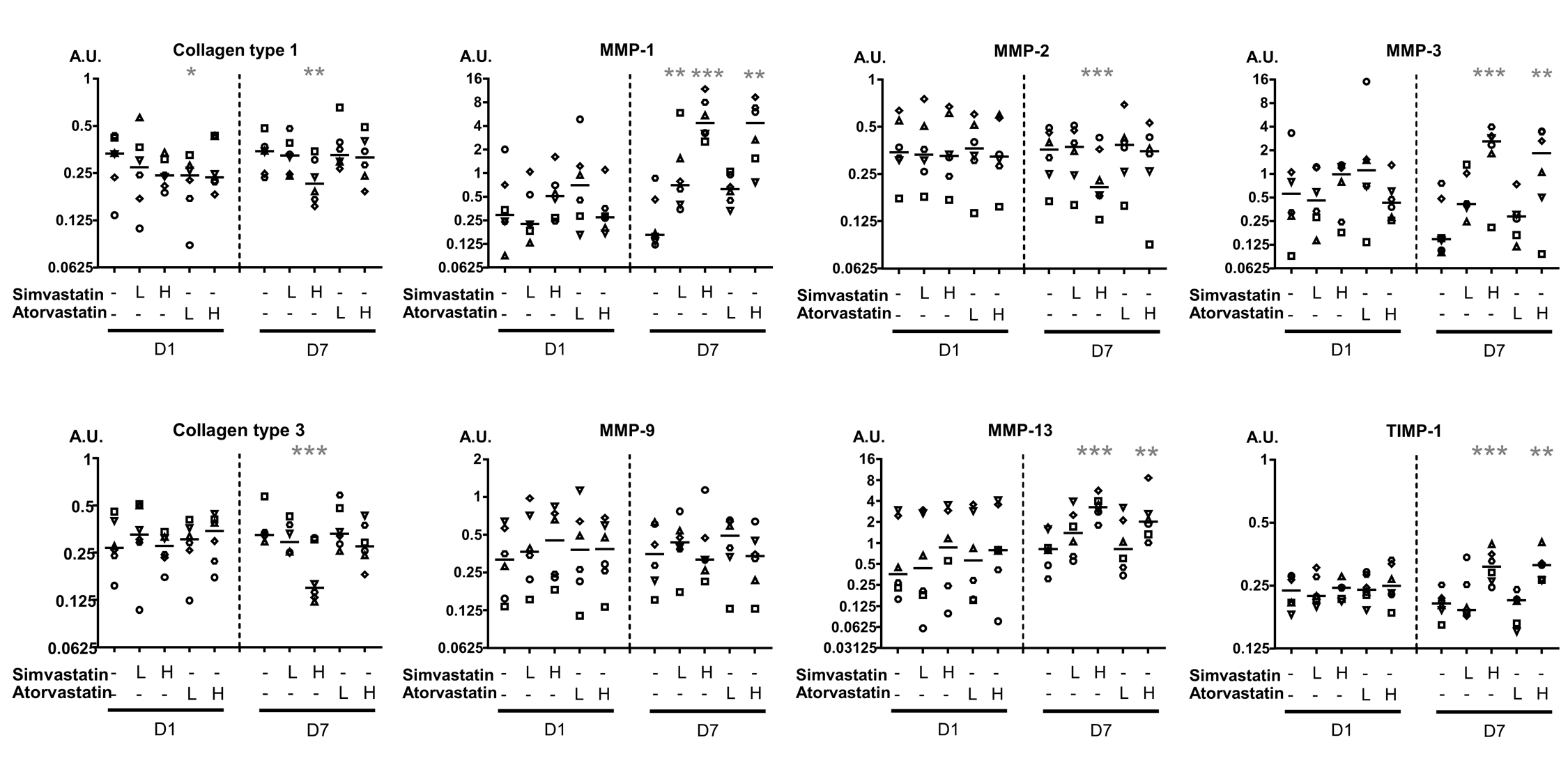

Supplement: S3 Fig — The gene expression after 1 or 7 days of statin treatment presented as the absolute values in arbitrary units (A.U.), which is a ratio between the mRNA and the housekeeping mRNA (RPLP0). Data is presented on a logarithmic y scale and the line represents the mean value. Individual donor-specific cell lines are represented by different symbols. Low dose (L) statin treatment corresponds to 0.05μM of either simvastatin or atorvastatin and high dose (H) corresponds to 0.5μM. Significant changes from DMSO-control are indicated by * (p<0.05), ** (p<0.01), and *** (p<0.001). (TIF) [file pone.0172797.s003.tif]
